# Supplementary material for: Are carbon emissions trading and green financial instruments synergistic? -Comprehensive quantitative research based on content analysis
Source: PLoS One. 2024 Mar 7;19(3):e0298601. doi: 10.1371/journal.pone.0298601 (PMC10919601; doi:10.1371/journal.pone.0298601)
Supplement: S1 Data — (ZIP) [file pone.0298601.s001.zip › Data/地级市Z_F工作报告.zip!/╦─┤¿/├α╤⌠╩╨/└┤╫╘╣1⁄2╓┌║┼í╛┬φ┐╦╩2╛▌═°í┐_2016.docx]

各位代表：

　　我代表市人民政府，向大会报告政府工作，请予审议，并请市政协各位委员提出意见。

　　2015年工作回顾

　　一年来，在省委、省政府和市委的坚强领导下，在市人大、市政协的监督支持下，我们牢牢把握稳中求进工作总基调，主动适应经济发展新常态，努力抢抓重大机遇，积极应对多重挑战，较好地完成了市六届人大六次会议确定的目标任务。全年实现地区生产总值1700.33亿元、增长8.6%，地方一般公共预算收入104.13亿元、增长2.34%，城镇居民人均可支配收入27170元、增长8.2%，农村居民人均可支配收入12349元、增长9.5%，居民消费价格上涨1.5%。

　　一、经济实现平稳增长

　　三次产业协调发展。“两个一号工程”深入实施。三次产业比重由15.7:51.0:33.3调整为15.3:50.5:34.2。传统农业向现代农业加快转变，实现农林牧渔业增加值265.06亿元、增长3.8%。绵阳国家农业科技园成功获批，江油市被确定为第三批国家现代农业示范区，安县成功创建国家农产品质量安全县。新增平武果梅等5个国家地理标志产品。新建成现代农业产业基地38个，高标准农田23.8万亩。工业经济平稳发展，规模以上工业增加值增长10.7%。新增规模以上工业企业81户。长虹控股集团销售规模突破千亿元大关，九洲集团工业总产值超过200亿元。市县两级设立3.5亿元企业应急转贷资金，为97户企业提供4.35亿元转贷资金支持。单位工业增加值能耗下降19.86%。淘汰江油市马角磷化等4户落后产能企业，淘汰化工落后产能4万吨、纺织落后产能1600吨。服务业发展势头良好，实现增加值581.35亿元、增长9.4%。实现电商交易额2232亿元，居全省第2位。三台县、北川羌族自治县获批全国电子商务进农村综合示范县，梓潼县被评为全国供销合作社电子商务示范县，涪城区被评为四川省唯一的全面创新改革（电子商务）试验区。金融机构人民币各项存款余额2882.91亿元、增长10%，贷款余额1532.85亿元、增长9.6%。仙海风景区、北川寻龙山景区成功创建国家4A级景区。入选首批中国研学旅游目的地。全市实现旅游总收入343.6亿元、增长23.9%。

　　投资消费稳定增长。完成全社会固定资产投资1154.09亿元、增长6.8%。完成工业投资354.24亿元、增长9%，技改投资327.43亿元、增长2.5%。基础设施投资294.59亿元、增长15.6%。民间投资707.95亿元、增长9%。159个省、市重点项目顺利实施，其中，马鸣风电场等42个项目顺利竣工投产，宝马N20发动机技术改造等51个项目加快建设，神华天明煤电一体化循环经济产业园等59个项目开工建设，皂角铺铁路货场扩建等7个项目前期工作有序推进。实现社会消费品零售总额879.16亿元、增长13%。成功举办中国（四川）电商发展峰会等110场展会，科博会蝉联中国十佳品牌会展项目。大力推动房地产促销售、去库存，商品房销售面积339.33万平方米、增长12%。

　　改革开放扎实推进。10大类166项改革事项基本完成。安县撤县设区方案上报国务院。非行政许可审批全面取消，“三证合一、一照一码”登记模式积极实施，新增各类市场主体3.34万户、增长3.71%。非公经济增加值首次突破千亿元大关，占GDP比重上升至60.1%。国企资产资源整合、分类管理等制度进一步完善。长虹控股集团市场化选聘总经理，九洲集团深化改革总体方案获批实施。绵阳市商业银行实现增资扩股。新增金融机构15家，新增富临精工等挂牌上市企业11户。农村改革持续推进，农村土地经营权流转96万亩、流转率23.5%。实施涪江流域水环境生态补偿和水质超标资金扣缴制度，出境断面水质全面达标。成功举办海科会、欧洽会绵阳专场等14场重大投资促进活动。涪城区跻身全国投资潜力百强区。新签约天津力神动力锂电池等项目330个，计划投资1556亿元。外贸进出口总额26.3亿美元。外事、台侨、口岸、海关、检验检疫等工作切实加强。被新华社评为“一带一路”最具活力城市。

　　二、科技城建设成效显著

　　创新平台加快建设。部际协调小组第12次会议成功召开，李克强总理、刘延东副总理分别对科技城建设作出重要批示和指示。成功纳入国家系统推进全面创新改革试验区域。科技城集中发展区启动区骨干道路主网基本成型，科技城大道连接线互通立交、八家堰大桥等重大基础设施项目全面完工。创新中心二期、软件产业园等创新平台功能逐步完善，核医学中心、军民融合产业创新联盟积极筹建。新增省级孵化器9家，孵化器总面积达到80万平方米。新增国家、省级企业技术中心7家，总数达到66家。建成国家、省级工程技术研究中心19个，国家、省级重点实验室25个。中国创新驿站绵阳工作站、中国技术交易所绵阳工作站作用有效发挥。全市实现技术合同成交总额7.5亿元。国家某重大专项选址绵阳工作取得积极进展。

　　创新要素加速聚集。国家自主创新示范区4项先行先试政策落地实施，全面推进大众创业万众创新的系列政策制定出台。科技与金融结合试点扎实推进，科技城军民融合金融服务中心挂牌运行。“千英百团”聚才计划深入实施，中国国际人才市场绵阳分市场成功获批。中国博士后西部服务团绵阳行活动顺利开展，中国创新创业大赛军转民大赛成功举办。“涌泉计划”成效明显，新增科技型中小企业2569户，新增29户成长为规模以上企业。专利申请7056件、增长26.1%，授权4367件、增长37.2%。研究与试验发展经费支出占地区生产总值比重6.53%，高新技术产业化指数79.6%，科技进步综合水平指数68.28%，被评为国家知识产权试点示范城市。

　　两新产业蓬勃发展。出台《关于加快引进培育高新技术产业和战略性新兴产业的意见》《工业重点产业及布局方案》，电子信息、新材料等八大重点产业加快发展。中物材料等71个两新产业重大项目顺利竣工，九洲北斗地基相位增强技术等关键技术取得重大突破。高新技术产业和战略性新兴产业产值占工业总产值比重分别为45.6%、34%，保持全省领先优势。出台军民融合企业标准认定暂行办法。新增军民融合企业83家、总数达446家，实现军民融合企业产值1720亿元、增长12%。

　　三、城乡区域协调发展

　　中心城区品质不断提升。城市建成区面积125平方公里，常住人口124.3万。城镇化率提高1.49个百分点，达到48%。135个市政设施重点项目完成投资31.9亿元。剑南路改造一期、安昌桥综合改造等工程建成投用。三江六岸主体景观带建设、九洲大道园林景观提升等绿化亮化美化工程加快实施，金家林收费站完成改造，城市家具色彩和户外广告设置方案编制完成。积极创建国家森林城市，城区空气达标率83.2%、较2014年提高7.2个百分点。三江湖湿地公园通过国家级评审。智慧城市建设有力推进，智慧交通、智能配电、数字城管初见成效。获批宽带中国示范城市。

　　城镇综合承载能力显著增强。成功入选第二批国家新型城镇化综合试点、第二批国家生态文明先行示范区。通过国家产城融合示范区评审。市县总体规划、控制性详细规划编制工作进一步规范，安县城市总体规划获省政府批复。三台县芦溪镇被评为全国美丽宜居小镇，游仙区魏城镇等国家、省重点小城镇加快建设，城镇基础设施建设完成投资80亿元。建成梓潼县文昌镇青龙村等416个幸福美丽新村，江油市二郎庙镇青林口村等4个村列入中国传统村落名录。

外联内通交通体系加快构建。交通建设大会战全面启动，完成交通投资47.7亿元。成兰铁路安县段加快建设。绵西高速全线开工，九绵高速、绵苍高速、平广高速前期工作扎实推进。G108线梓潼绕城改线、江油中坝至大堰快速通道等道路建成通车，G247线江油至北川段、三台至射洪段和G245线盐亭至南部段等国省干线公路大中修工程顺利完成。79座国省干线病危桥完成整治。建设农村公路1646公里，100%的乡镇通油路和水泥路，100%的村通公路，89%的村通硬化路。绵阳机场新增通航城市7个、总数达26个，年旅客吞吐量154.8万人次。

四、社会民生持续改善

　　民生实事全力推进。十项民生工程扎实开展，16件民生大事全部落实，投入资金132.57亿元、增长6%，被新华网评为首批全国民生改善十佳典范城市。新增城镇就业5.4万人，城镇登记失业率3.9%。开工保障性住房和危旧房棚户区改造13692套，竣工7052套，改造农村危旧房3315户。发放城乡低保资金5.7亿元，帮助困难群众331.7万人次。实施80周岁以上老人高龄津贴制度，惠及14万老人。实施农村义务教育学生免费午餐计划，惠及12.7万学生。下调社会保险缴费基数和费率，为企业和参保者减轻负担4.7亿元。各类社会保险参保579万人次，发放资金111.5亿元。新型农村合作医疗提标扩面。脱贫攻坚深入实施，5.11万人实现脱贫，三台县丘区农村脱贫攻坚示范区建设取得阶段性成效。对口支援阿坝州壤塘县、红原县工作扎实开展，援建雅安市雨城区灾后重建任务圆满完成。

　　社会事业健康发展。高考本科硬上线率、本科硬上线万人比连续15年全省“双第一”，重点本科录取人数首次突破万人。7个县市区被认定为国家义务教育发展基本均衡县。全国第九届残运会暨第六届特奥会成功举办,三台县被命名为首批全国青少年校园足球试点县。央视中秋晚会、李白诗歌奖颁奖典礼圆满举行。单独二孩政策有序实施，人口自然增长率2.09‰。顺利通过国家卫生城市复审，被评为全国基层中医药工作、全国防震减灾工作先进单位，保持全国文明城市称号。广电、科普、史志、档案、统计、气象、水文、测绘等工作切实加强。民族、宗教、工会、青少年、妇女儿童、残疾人、慈善、老龄等事业健康发展。

　　社会治理不断提升。平安绵阳建设持续推进，“六五”普法圆满完成，村（社区）法律顾问实现全覆盖，涉法涉诉信访纳入法治轨道。安全社区和企业安全生产标准化建设切实加强，未发生重特大安全事故。食品安全状况满意度连续三年全省第一。违法建设整治扎实推进，拆除违法建筑1.12万宗，整治面积142万平方米。应急管理体系更加健全。国防动员、国防教育持续深化，后备力量建设得到加强，被评为第十届四川省双拥模范城。国安、人防、保密、邮政、住房公积金等工作积极推进。

　　五、依法行政深入推进

　　法治政府加快建设。出台《绵阳市人民政府议事规则》《绵阳市重大行政决策程序规定》，规范性文件合法性审查严格落实，依法行政决策机制进一步完善。政府权力清单公布推行，行政执法裁量权逐步规范。建立市政府与市人大常委会、市政协定期联系机制，完善政府部门与民主党派对口联系制度。237件人大代表建议、370件政协委员提案全部办结。

　　政务服务不断优化。行政审批“江油模式”全面推广，市本级办结行政审批事项18.9万件、办理提速94.7%。公共资源交易管理制度进一步完善，工程建设项目招投标、土地矿权出让等5大类109个项目陆续纳入公共资源交易平台。20个市级部门热线并入市长热线。“绵阳城事”微信公众平台荣获全国政务微信卓越奖。

　　工作作风明显转变。“三严三实”专题教育扎实开展，政府系统文件、会议进一步减少。85个市级部门预算全部公开，“三公”经费下降10.5%。“三走遍一深入”活动持续推进，解决了一批群众关心的热点、难点问题。审计监督和监察问责力度加大，政府系统政纪处分102人。

　　各位代表，需要特别说明的是，受经济下行压力持续加大等因素影响，地方一般公共预算收入、进出口总额、城镇居民人均可支配收入、农村居民人均可支配收入4项指标未能完成预期目标任务。

　　“十二五”发展主要成就

　　“十二五”时期，面对严峻复杂形势，我们团结带领全市各族人民克难奋进，圆满完成“5·12”汶川特大地震灾后重建任务，经济社会发展取得了重大历史性成就。

　　综合实力显著增强，主要经济指标全省领先。地区生产总值年均增长11.2%，高于全省0.4个百分点。人均地区生产总值从20053元增加到35754元。全社会固定资产投资、社会消费品零售总额年均增长7%、15.2%，地方一般公共预算收入年均增长18.2%。农业增加值年均增长3.8%；全部工业增加值年均增长14.4%，电子信息、汽车等八大优势产业进一步壮大；服务业增加值年均增长9.7%，总量居全省第2位。

　　科技城建设成效显著，创新发展位居西部前列。“五地”建设成果丰硕，科学新城、空气动力新城、航空新城建设取得重大进展。绵阳经开区升级为国家级经济技术开发区。科技城集中发展区加快建设。军民融合企业产值占工业总产值比重超过60%。科技型中小企业数量累计增加6108家。万人有效发明专利拥有量年均增长27%，高新技术产业产值年均增长13.2%。获批全国科技进步先进市、全国首批科技与金融结合试点市。

　　改革开放纵深推进，发展活力充分迸发。启动改革试点79项，16项改革试点被列为全国、全省改革案例。非公有制经济活力显著增强，增加值占比提高6.9个百分点。财税改革平稳推进，“营改增”试点扎实开展。金融改革有序实施，农村信用社改革全面推进，金融机构总数达190家，增加47家。上市挂牌企业24家，增加17家。新引进世界500强、国内500强企业16家，总数达到25家。国际友好城市达到22个，与国内重点区域和城市合作全面加强。

　　县域经济发展加快，城乡面貌日新月异。涪城区连续四年跻身全省十强县前三甲，江油市经济总量突破300亿元大关，三台县、高新区经济总量超过200亿元。全市城镇化率提高8.15个百分点，17.2%的村建成幸福美丽新村。交通基础设施累计投资289.08亿元，新改建公路7073公里，成绵乐城际铁路、成绵高速复线、成德南高速、绵遂高速等交通干线建成通车。绵阳机场年旅客吞吐量增加97.1万人次。水利设施累计投资132.25亿元，新增有效灌溉面积37.7万亩。武都水库顺利建成，入选国家级水利风景区。

　　社会事业全面发展，人民幸福指数提高。累计安排民生工程资金570.83亿元。城镇、农村居民人均可支配收入分别年均增长12.2%和13.7%。城镇新增就业累计23.7万人，城镇登记失业率控制在4%以内。社会保障水平进一步提升。减少贫困人口59.3万人，贫困发生率下降到3%。148.3万农村人口饮水安全问题得到解决。义务教育全面实行“三免一补”，基础教育质量领跑西部。高等院校达到14所,居全国地级市前列。电影《兰辉》获全国“五个一”工程奖。国家智慧城市试点、国家文化和科技融合示范基地加快建设。医疗资源总量西部领先，国家“十二五”重大传染病防治示范区建设顺利完成。森林覆盖率从48.99%提高到52.4%。主要污染物总量减排目标全面完成。县乡公路危险路段全部安装安全防护栏。人民群众对社会治安满意度连续五年名列全省前2位。

　　各位代表！五年来，我们着力加强政府自身建设，主动接受人大法律监督、工作监督和政协民主监督，累计办理986件人大代表建议和1973件政协委员提案。85项行政审批事项调整取消，四级政务服务体系全面建成。政府绩效考核管理切实加强，党风廉政建设和反腐败工作取得新成效。

　　五年来取得的巨大成就，是党中央、国务院，省委、省政府坚强领导和亲切关怀的结果，是市委正确领导，市人大、市政协和人大代表、政协委员监督支持的结果，是全市广大干部群众奋力拼搏的结果。在此，我代表市人民政府，向全市各族人民，向人大代表、政协委员，向各民主党派、工商联、无党派人士和各人民团体，向所有关心支持绵阳改革发展的同志们及社会各界人士，表示诚挚感谢！向驻绵解放军指战员、武警官兵、政法干警和民兵预备役人员，表示诚挚感谢！向港澳台同胞、广大侨胞和海内外朋友们，表示诚挚感谢!

　　我们也清醒地认识到，全市经济社会发展还面临不少困难和问题，主要表现在：发展不足、发展质量不高、发展不平衡的最大市情仍然没有根本改变；科技城建设市场化程度不高，军民融合体制机制障碍尚未根本突破；部分行业和企业生产经营困难，亏损面扩大，融资难、融资贵问题仍然突出，财政收入增长放缓；基本公共服务均等化水平有待进一步提高，城乡居民生产生活条件尚需进一步改善；脱贫攻坚任务依然艰巨，还有约12万贫困人口仍未脱贫；政府行政能力和水平还需要进一步提升。对这些问题，我们将高度重视，认真解决。

“十三五”时期总体目标任务

　　“十三五”时期是全面建成小康社会的决胜阶段。根据党的十八届五中全会、省委十届七次全会、市委六届十次全会精神和市委关于“十三五”规划编制的建议，市政府组织编制了《绵阳市国民经济和社会发展第十三个五年规划纲要（草案）》，现提请大会审议。这里简要说明如下：

　　“十三五”时期经济社会发展的总体目标：经济发展水平迈上新台阶。经济保持中高速增长，增速8%以上，总量2450亿元左右，提前实现地区生产总值和城乡居民人均收入比2010年翻一番。县域经济发展加快，城镇化水平显著提升。科技城建设取得新突破。基本建成国家军民融合创新改革示范区、创新驱动发展特色示范区和西部经济发展新兴增长极。科技进步综合水平指数达70%，科技城研究与试验发展经费支出占地区生产总值比重达6.8%，万人有效发明专利拥有量达8件。人民幸福指数得到新提升。城乡居民人均收入高于全省平均水平,增长速度高于经济增速，提前实现现行标准下贫困人口全部脱贫。社会文明程度实现新提高。人民思想道德素质、科学文化素质、健康素质明显提高，全社会法治意识不断增强，诚信体系逐步健全，公共文化服务体系更加完善。重点领域改革取得新进展。全面创新改革试验区建设取得重大突破，形成一批可复制、可推广的改革经验和重大政策，创新创业生态体系和开放型经济新体制基本形成。生态文明建设取得新成效。生态环境持续改善，森林覆盖率达53%，单位GDP能耗、主要污染物排放和二氧化碳排放达到省控目标。“十三五”时期经济社会发展的基本要求：一是坚持以人民为中心的发展思路；二是坚持把发展作为第一要务，把经济建设作为兴市之要；三是坚持深化改革扩大开放；四是坚持以法治思维和法治方式推动发展；五是坚持党的领导。“十三五”时期经济社会发展的主要任务：一是创新驱动，推动国家科技城突破性发展；二是转型升级，促进产业发展迈向中高端；三是改革开放，增强经济社会发展动力活力；四是互联互通，推进现代基础设施体系建设；五是优化布局，统筹城乡区域发展；六是以人为本，共享经济社会发展成果；七是绿色发展，打造生态文明新家园。

　　各位代表，我们坚信，经过今后五年努力，绵阳这座充满生机、充满活力的城市，经济将更加发达、科技将更加进步、民主将更加健全、文化将更加繁荣、人民生活将更加殷实、生态环境将更加优美，必将以崭新的姿态屹立在中国西部大地！

　　2016年工作安排

　　2016年是我市实施“十三五”规划的开局之年。做好今年政府工作，要全面贯彻党中央、国务院，省委、省政府和市委决策部署，认真践行“五大发展理念”，主动适应经济发展新常态，把握稳中求进工作总基调，坚持科学发展、加快发展，突出全面创新改革牵引，着力推进供给侧结构性改革，提高经济增长质量和效率，统筹抓好稳增长、调结构、惠民生、防风险，努力实现“十三五”发展良好开局，加快建设国家科技城和幸福美丽绵阳。

　　2016年经济社会发展主要目标：地区生产总值增长8%，规模以上工业增加值增长10%，全社会固定资产投资增长8%，社会消费品零售总额增长11.5%，地方一般公共预算收入同口径增长8%，城镇和农村居民人均可支配收入分别增长8%以上、9%以上，城镇登记失业率控制在4.3%以内，居民消费价格指数调控目标与全省一致，完成省政府下达节能减排和环保目标任务。

　　为实现上述目标，今年将重点抓好以下工作：

　　一、着力扩大投资消费，千方百计稳定经济增长

　　坚持以项目为中心组织经济工作，狠抓有效投资，扩大消费需求，推动经济持续健康发展。

　　大力开展“项目年”行动。精准对接国家、省重点投资领域和专项建设基金投资方向，包装储备一批重大项目，争取更多项目纳入国家、省上大盘子。建立上下联动的项目协调推进机制，完善领导联系项目制度。突出抓好75个省重点项目(含跨区域及全省打捆项目33个)、160个市重点项目和249个县市区（园区）重点项目。做好绵苍高速、中经云大数据光存储产业中心等67个项目前期工作，推动九绵高速、B11等214个项目开工建设，促进川汽新能源乘用车生产基地、神华天明煤电一体化循环经济产业园等203个项目加快建设，实现生活垃圾焚烧发电等157个项目竣工投产。

　　着力增加有效投资。突出抓好工业投资，用好产业发展投资引导基金和企业应急转贷资金，新开工工业和技改项目100个以上、竣工105个以上，实现工业投资375亿元以上、技改投资345亿元以上。大力推进交通、能源、信息、水利、市政等基础设施建设投资，完成投资325亿元以上。深化投融资体制改革，推动项目审批便利化，放宽民间资本准入限制。充分发挥PPP投资引导基金作用，推进一批政府与社会资本合作项目签约落地，实现民间投资790亿元以上。力争全年完成全社会固定资产投资1246亿元。

　　稳步扩大消费需求。以新消费引领新供给、打造新动力，增加中高端、多元化服务供给，培育健康养老、文化、旅游等消费热点。加快建设京东绵阳特色线下体验馆、天泰进口商品直销中心等项目。健全农村流通网络，新建区域性农资配送中心1个、县级配送中心2个。着力打造北川羌绣、梓潼蜜柚、三台麦冬等特色产品。继续办好“绵阳国际汽车展”等展会，组织企业参加“惠民购物全川行”“川货全国行”等活动。认真落实带薪休假制度，鼓励错峰休假、弹性作息。稳妥推进住房制度改革，积极引导消费预期，继续化解房地产库存。

　　二、推进全面创新改革，开创科技城建设新局面

　　系统推进全面创新改革试验，提升全社会创新、创业和创造能力，增强经济发展动力活力和核心竞争力。

　　扎实推进全面创新改革试验。组织实施好全面创新改革总体方案和年度计划，探索军民融合深度发展科学化、法治化、规范化路径，着力形成一批可复制、可推广的改革经验和重大政策。联合驻绵科研院所和十大军工集团实施一批军民融合特色产业园区和产业项目。组建军民融合产业发展基金。推动院地军地共建技术研发平台、产业技术创新联盟。用好国家军民两用技术交易中心、中物院技术转移中心、四川省军民融合研究院等军民融合平台。建立军民两用技术成果库。鼓励银行金融机构和社会资本参与军工企业股份制改造。开展国防科研院所企业化改革，鼓励符合条件的军工企业上市。开展军民融合企业认定试点。力争军民融合企业产值突破1900亿元。

　　全力推动双创工作。积极申建国家小微企业创业创新基地城市。大力发展众创、众包、众扶、众筹空间，推动孵化器、加速器市场化、专业化建设和运营，启动建设创新中心三期、欧盟国际企业孵化器，科技企业孵化器达到50家以上，总面积突破100万平方米。加快建设科技创新服务中心线上平台"优科服"、创投集聚平台和人才集中服务平台，建好中国国际人才市场绵阳科技城分市场。新建省级重点实验室、省级工程技术研究中心各1家。设立科技型中小微企业发展引导基金。大力实施"涌泉计划"和科技型中小企业提质增效工程，新增科技型中小企业2000户以上、新增升规入统企业60户以上。实施高新技术企业培育计划，新增高新技术企业50户以上，实现总产值1500亿元。全面推进国家知识产权试点示范城市建设，申请专利8000件，其中发明专利3300件，新增专利实施项目1500个、新增产值190亿元，实现技术合同成交总额8亿元以上。

　　狠抓重点领域改革。加强供给侧结构性改革，制定去产能、去库存、降成本工作推进方案。深化农业农村改革，以放活土地经营权为重点，按期完成土地承包经营权确权颁证，有序推进供销社改革。加强国资国企改革，加快完善现代企业制度，调整国有资本布局结构，推进产权多元化，完善国有资产管理体制。推进财税体制改革，健全规范透明的预算制度，转变财政支持发展方式，构建财政金融互动政策体系，加强政府债务风险管控。深化投融资体制改革，用好城市基础设施建设投资引导基金等5支基金。加快金融改革步伐，推进商业银行和农村信用社改革，支持企业上市融资，新增上市挂牌企业10家、上市辅导企业100家。毫不动摇地支持非公有制经济加快发展。

　　提升园区发展水平。围绕《科技城发展规划（2016-2020年）》《科技城集中发展区总体规划》，编制完善集中发展区核心区控规及城市设计导则。积极打造集中发展区，加快建设总投资447亿元的48个重大项目。支持高新区、经开区和科创区率先发展，推动三台工业园、游仙经济开发区等8个省级园区错位发展。制定特色园区培育方案，推动园区专业化、高端化、特色化发展，加快建设四川绵阳汽车产业园、北川通航产业园、长虹智慧制造产业园等专业化产业园。园区工业经济占全市工业经济比重80%以上。

三、加速产业转型升级，加快构建现代产业体系

　　深入实施“两个一号工程”，努力优化三次产业结构，打造特色优势产业集群，提高产业实力和可持续发展能力，加快构建现代产业体系。

　　推动工业提质增效。着力工业强基，加快发展高新技术产业和战略性新兴产业，产值占工业总产值比重分别达46.5%、35%。支持食品、化工等优势产业产品结构调整，加速冶金、建材等传统产业改造升级。逐步化解过剩产能，淘汰5户以上企业落后产能。稳妥处置“僵尸企业”，多兼并重组、少破产清算，同步做好职工安置工作。扎实开展降成本增效益专项行动，降低企业生产经营成本和税费负担。实施大企业大集团培育计划，新增100亿元企业1户、50亿元企业2户，4亿元以上企业总数达100户。支持中小微企业特色发展，新增“专精特新”企业15户、“小巨人”企业10户，力争新增规模以上工业企业100户，构建大企业“顶天立地”、小企业“铺天盖地”的企业发展格局。加大品牌培育力度，新增国家驰名商标1个，省著名商标2个。实施质量对标提升行动，建设国家电器安全质量监督检验中心，创建国家质量强市示范城市。

　　加快发展现代服务业。实施“互联网+”制造试点示范行动，发展云计算、大数据产业。开展“全企入网、全民触网、电商示范”三大工程，争创国家电子商务示范城市，培育年交易额超100亿元电商企业3家，电商交易额超过2400亿元，网络零售交易额超过100亿元。深化科技金融结合试点，加快全国小微企业信用体系试验区建设，积极打造金家林金融集聚区。深入推进高新区全国首批科技服务业试点。新增科技服务企业10家。加快建设科技城物流产业园，打造川西北重要的物流集聚区和集散地。大力发展现代旅游业，重点推动王朗、虎牙等旅游景区项目开发，加快培育阳光度假、乡村休闲等旅游业态，创建4A级景区2个，实现旅游收入420亿元。着力发展文化创意产业，力争文化产业增加值达到120亿元以上。

　　转变农业发展方式。全力推进农业转方式调结构，大力发展生态循环农业，促进粮经饲统筹、农林牧渔结合、种养加一体、一二三产业融合发展，提升农业集约化、专业化、组织化、社会化水平。加快建设农村产权交易中心，积极培育新型职业农民，大力发展农村新型经营主体，新增国家农民合作示范社5家、家庭农场200家。加大农田水利、农机作业配套设施等建设支持力度，新建现代农业产业基地13个、高标准农田26万亩，推进武引灌区二期、草溪河水库和小微水利设施等水利工程建设。做好第三次全国农业普查工作。

　　四、实施三大攻坚行动，着力建设美丽宜居绵阳

　　实施城建、交通、集中发展区建设攻坚行动，整体提升城市承载能力和功能品质，加快建设智慧城市，让市民生活更方便、更舒心，城市更宜居、更美丽。

　　大力推进城建攻坚行动。坚持“五个统筹”，积极推进城市建设工作。启动城市总体规划修编前期工作，推动城市规划与“十三五”国民经济社会发展规划、土地利用总体规划“多规合一”，完善一主四副、多组团城市格局。加快城市轨道交通项目前期工作。启动重构一环、贯通二环道路建设工程，改造磨家高速收费站，完成涪江二桥维修加固等工程。优化城市综合交通体系，加密市域公交线网，新建魏城公交枢纽站等5处公交场站。有序推进城市地下空间开发，新建中心城区停车场2个。推进剑门路西段片区排水改造等供排水设施建设。大力实施城市绿化亮化美化，加快三江六岸主体景观带建设，改造五一广场等城区公园，新增公园绿地70万平方米。优化智慧城市顶层设计方案，有序实施智能交通、智能电网、数字城管等智慧城市建设项目。统筹政府、社会、市民三大主体，推动城市管理重心下移、职能下沉，促进政府治理与社会自我调节、居民自治良性互动。

　　深入实施交通攻坚大会战。开展绵遂铁路前期工作，加快成西客专江油段、成兰铁路安县段、火车货站迁建等项目建设。抓好绵苍高速、绵中高速、平广高速、三大高速等项目招商引资工作，全线开工建设九绵高速，加快推进绵西高速建设。实施国省干线改造升级，加快建设G108线绵阳城区过境改线、G245线三台段、G247线江油至平武段，以及江油至三台芦溪快速通道(原S205线绵阳城区改线)、S416线凤凰立交至中江段、S418线安县至雎水快速通道，全面完成国省干线病危桥梁整治工程。积极推进县乡公路改造，改善县乡道400公里、村道600公里，实施渡改桥5座。完善绵阳机场设施设备，启动扩建停机坪等工作。完成交通建设投资100亿元以上。

　　实施科技城集中发展区基础设施建设攻坚行动。推进科技城大道河边段、启动区1、2、4号道路等11条道路建设。加快地下综合管廊规划设计，确保一期工程年内开工建设。加快海绵城市规划编制，努力争创海绵城市试点。积极推进安昌河、草溪河流域综合治理，启动河堤水下项目建设。加大河边镇安置房及场镇二期改造力度，做好拆迁群众安置工作。

　　五、深化对内对外开放，切实增强经济发展活力

　　统筹推进高水平双向开放，构建开放型经济新体制，形成全方位开放新格局。

　　提升投资促进实效。强化政府部门联动招商，选聘海外招商专员，充实驻外招商力量。突出环渤海、长三角、珠三角和欧美、日韩等重点区域，持续开展精准专业招商。继续办好科博会，积极参加全球川商返乡发展大会、中外知名企业四川行、中国西部投资说明会、中国西部海外高新科技人才洽谈会等活动。围绕产业链、创新链招大引强、招新引优、招才引智，力争引进5亿元以上重大产业项目超过40个，到位国内省外资金410亿元以上、到位资金额进入全省前三，到位外资1.2亿美元。

　　加快走出去步伐。创新外贸发展模式，实施“自主品牌出口增长”计划和“优进优出”战略。鼓励企业参与“一带一路”建设，借助“蓉欧快铁”“中亚班列”开拓国外市场，扩大电子信息、节能环保等高新技术产品出口，力争实现外贸进出口21.5亿美元以上。积极参与国际产能合作“111”工程，推动富余产能向境外转移。参加“万企出国门”“千企行丝路”活动，鼓励长虹、九洲等企业通过并购重组、合资合作等方式开展跨国经营，实现对外投资总额1.2亿美元。

　　全面加强区域合作。加快出口加工区升级改造，启动建设综合保税区基础设施，加快跨境电商产业园建设，建好中德产业创新合作平台，全力打造对外开放、区域合作载体。加强与成都、德阳在区域协同、交通建设、科技金融服务等方面合作，努力实现平台共建、资源共享、政策共用，助推成德绵一体化发展，打造成德绵协同创新共同体。密切与成渝经济圈、长江经济带联系，深化与港澳台、国内外友好城市合作交流。

　　六、努力壮大县域经济，促进区域均衡协调发展

　　按照因地制宜、分类指导，统筹兼顾、重点突破的基本思路，推动县市区协调联动、错位发展，形成区域协调、城乡统筹发展格局，夯实绵阳经济底部基础。

　　提高县域经济实力。认真落实省委省政府支持县域经济发展相关政策，制定加快县域经济发展指导文件。积极推动纳入市辖区和重点开发区的涪城区、游仙区、江油市、安县优化要素配置，加快建设先进制造业和现代服务业强县，率先实现城乡一体化发展。大力支持纳入农产品主产区的三台县、梓潼县、盐亭县稳定粮食生产，加快建设现代农业强县，因地制宜发展工业和服务业，不断优化产业结构，推动转型升级。着力引导纳入重点生态功能区的平武县、北川羌族自治县，切实加强生态保护，优先推动旅游产业发展，建设旅游强县，推动形成特色鲜明的区域经济板块，构建差异化、特色化、可持续发展格局。

　　加快建设新型城镇。坚持以人的城镇化为核心，实施居住证制度，推进基本公共服务由户籍人口向常住人口覆盖，促进农业转移人口市民化，城镇化率提高1.5个百分点。稳步推进行政区划调整。加强污水、垃圾处理等基础设施和公共服务设施建设，努力完善城镇功能，建设宜业宜居城镇。落实“百镇建设试点行动”，加快建设涪城区龙门镇、三台县西平镇等21个国家和省级试点示范镇。

　　打造幸福美丽新村。积极开展农村电网升级，实现边远山区、民族地区农村电网全覆盖。大力实施国家电信普遍服务试点，实现全市行政村100%通宽带。保护和传承历史、地域文化，大力发展乡村文化、民俗文化，促进农旅、文旅融合，建设旅游精品村寨20个。抓好北川羌族自治县、梓潼县、平武县第三轮省级幸福美丽新村示范县建设，打造“小规模、组团式、微田园、生态化”新农村综合体，建设550个业兴、家富、人和、村美的幸福美丽新村。

七、切实加强环境保护，努力建设生态文明家园

　　树立尊重自然、顺应自然、保护自然的理念，促进人与自然和谐共生，让绵阳天更蓝、地更绿、水更清、人更美。

　　构建绿色生态屏障。严格划定和管控生态保护红线，推进天保工程二期、退耕还林建设。营林造林20万亩，森林覆盖率提高到52.6%，争创国家森林城市。加强自然保护区建设，实施野生动植物抢救性保护工程，建立大熊猫碳汇基金，争取中国大熊猫国家公园尽快挂牌。加快建设三江湖国家湿地公园、凤凰山森林公园。完善涪江流域水环境生态补偿和水质超标资金扣缴制度，全面推行县级党政主要负责人自然资源资产和环境责任审计，抓好生态环境损害赔偿制度试点。

　　加大环境治理力度。深入开展大气、水、土壤污染防治。完善重污染天气预警体系，以“减排、压煤、抑尘、治车、控秸”为重点，推动大气质量改善，力争空气质量优良天数达到85%以上。加大饮用水源和良好水体保护力度，持续推进芙蓉溪、木龙河等小流域综合治理和黑臭水体整治。加强涉重金属、危险废物企业监管力度，推动跨区域联防联控。实施工业污染源全面达标排放计划，加强农业面源污染治理。开展城乡环境综合整治“五大工程”，全面提升环境质量。

　　促进资源高效利用。推广使用风电、太阳能等清洁能源，推动形成绿色生产方式和消费方式。发展绿色低碳循环经济，加快中科循环经济产业园等环保项目建设，促进废水、废气、建筑垃圾等资源综合治理。加快企业节能降耗技术改造，控制高耗能高污染行业增长，确保完成省政府下达的单位GDP能耗下降目标。实施能源和水资源消耗总量强度双控行动，推进城镇低效用地再开发和工矿废弃地复垦，促进资源节约和高效利用。

　　八、深入实施脱贫攻坚，加快补齐小康社会建设短板

　　将脱贫攻坚作为今年的重点任务和第一民生工程来抓，突出打好“3+10”组合拳，确保3.6万贫困人口、86个贫困村“摘帽”。

　　夯实脱贫攻坚工作基础。编制完成“十三五”扶贫总体规划和专项规划，制定实施10个专项扶贫方案年度计划。认真开展贫困人口信息数据对比、排查核实，全面完成贫困人口规模、致贫原因、扶持举措等信息入库，实现“市有卷、县有档、乡镇有簿、村有册、户有卡”。积极开展贫困统计监测，加强贫困动态管理，对脱贫的及时销号，对返贫的及时挂号，实现扶贫台账与脱贫台账相衔接、相对应、可追溯，努力提高扶贫质量。

　　扎实推进精准脱贫工程。以三大片区为主战场，把“六个精准”“四个一批”要求真正落实到村、到户、到人头。扶持生产和就业发展一批，坚持“一乡一业、一村一品”，支持贫困村、贫困户因地制宜发展种养业和传统手工业。加大贫困户子女接受教育资助力度，阻断贫困代际传递。生态扶贫和移民搬迁安置一批，分类引导群众从生态敏感区、地质灾害隐患点，向县城、重点镇和新村搬迁。低保政策兜底一批，逐步提高农村低保标准，加快低保线与脱贫线“两线合一”，稳妥实行农村“三无”特困人员集中供养。医疗救助扶持一批，提升贫困人口新农合筹资标准，提高大病报销金额，加大医疗救助、慈善救助等帮扶力度。统筹解决好插花式贫困问题。

　　着力创新脱贫攻坚机制。认真执行脱贫攻坚目标、任务、资金、权责“四到县”制度，扎实开展驻村帮扶，深化市级部门对口帮扶，做到分工明确、责任清晰、任务到人、考核到位。鼓励民主党派、群团组织、企业高校、科研院所，通过爱心捐赠、志愿服务等形式参与扶贫，形成精准扶贫、精准脱贫的强大合力。构建扶贫资金多元投入机制，加大财政扶贫投入力度，引导更多金融资金和社会资本参与扶贫开发。

　　九、切实保障改善民生，积极提升社会治理水平

　　按照“守住底线、突出重点，完善制度、引导舆论”的思路做好民生工作，大力发展社会事业，努力让全市人民在改革发展中有更多获得感。

　　全力办好民生实事。持续增加民生投入，抓好十项民生工程，办好20件民生大事。实施更加积极的就业政策，鼓励大学生、返乡农民工、下岗职工等重点群体以创业带动就业，新增城镇就业4.5万人以上。继续推进棚改货币化安置，开工保障房和危旧房棚户区改造1.17万套、基本建成1.2万套。建立更加公平可持续的社会保障制度，提高社会保障水平，有序推进机关事业单位养老保险改革。加快城乡居民医保整合，实现大病保险全覆盖。落实农村义务教育学校学生免费午餐计划。完善社会救助体系，加强对特殊困难群体的精准帮扶，保障困难群众基本生活。继续做好支援阿坝州壤塘县、红原县工作。

　　大力发展社会事业。建立健全全国文明城市建设常态化机制。推进基本公共教育服务均等化，力争县市区全部通过义务教育发展基本均衡县认定。提升高中教育质量，加快现代职业教育发展，开工建设公办幼儿园13所。关爱农村留守儿童、留守妇女、留守老人。精心办好全市第六届运动会，大力开展校园“三大球”活动。积极发展文化事业，完成绵阳博物馆新馆建设，加快构建现代公共文化服务体系。实施哲学社会科学创新工程。推进“健康绵阳”建设，加强医疗、医保、医药“三医”联动，启动城市公立医院改革，加快区域医疗联合体建设。积极发展中医药事业，加强公共卫生和重大疾病防控。做好计划生育服务管理，稳妥有序实施全面两孩政策。完善以居家为基础、社区为依托、机构为补充的养老服务体系，初步构建医养结合、全域覆盖的养老服务格局。

　　努力创新社会治理。开展信访“三无”创建活动，推动信访积案化解。全面启动“七五”普法，深化“法律七进”。创新社区治理，实施“三社”联动。积极支持工会、共青团、妇联等群团组织参与社会治理。完善立体化治安防控体系，依法严厉打击各类违法犯罪行为。加强安全生产工作，深化重点领域安全监管,建成2个国家级、10个省级安全社区。建立严密高效、社会共治的食品安全治理体系，创建国家食品安全城市。做好地震、气象、水文监测预报和地质灾害防治。强化金融风险预警预测，依法解决涉众性借贷和非法集资问题，守住不发生系统性、区域性金融风险的底线。开展民族团结进步创建活动，推进宗教事务依法管理。加强国防动员和后备力量建设，支持军队改革，做好双拥工作。提高突发事件应急处置能力，确保城市安全、市民安宁、社会安定。

　　十、坚持为民务实清廉，狠抓政府系统自身建设

　　坚持依法行政、务实高效、清正廉洁，切实增强政府执行力和公信力，建设人民满意政府。

　　推进依法行政，建设法治政府。把政府工作全面纳入法治轨道，严格执行市人大决议决定，自觉接受市人大及其常委会法律监督和工作监督，主动接受市政协民主监督，认真办理人大代表建议和政协委员提案。进一步完善权力清单、责任清单和负面清单。健全决策咨询制度，提高民主决策、科学决策、依法决策水平。广泛听取民主党派、工商联、无党派人士，以及工会、共青团、妇联等人民团体意见，高度重视社会监督和新闻舆论监督。

　　转变政府职能，建设服务政府。减少发文和会议数量，严控各类检查、培训、评比表彰活动。推进简政放权，巩固扩大“五个再砍掉一批”成果。加快政务大厅功能升级，全面实施“先照后证”，切实加强事中事后监管，把“放、管、服”改革引向深入。推动电子政务建设，拓展网上办事大厅功能，让群众少跑路、少花钱、少费时。加大财政预算、公共资源配置等方面信息公开力度。

　　加强反腐倡廉，建设廉洁政府。严格落实党风廉政建设各项规定，巩固党的群众路线教育实践活动、“三严三实”专题教育成果，持之以恒推进正风肃纪，防止“四风”问题回潮反弹。严格“三公”经费管理，完成机关公务用车改革。强化土地出让、工程建设等重点领域监管和审计监督，切实加强行政权力监督制约。

　　各位代表！功崇惟志，业广惟勤。让我们紧密团结在以习近平同志为总书记的党中央周围，在省委省政府和市委坚强领导下，紧紧依靠全市各族人民，开拓创新、拼搏实干，为加快建设国家科技城和幸福美丽绵阳而努力奋斗！
